# Robust Rear-View Human Tracking for Robotic Visual Sensing: A Spatiotemporal Prediction and Multi-Modal Fusion Approach

**DOI:** 10.3390/s26092884

**Published:** 2026-05-05

**Authors:** Xu Jia, Jia Xie, Yongguo Li, Jintao Liang, Zengmin Zhang

**Affiliations:** 1 School of Engineering, Shanghai Ocean University, Shanghai 201306, China; jiaxu666946@gmail.com (X.J.);; 2 School of Mechano-Electronic Engineering, Xidian University, Xi’an 710071, China

**Keywords:** vision sensors, robotic tracking, rear-view human tracking, adverse weather, spatiotemporal prediction, Kalman filter, multimodal fusion

## Abstract

**Highlights:**

**What is the main finding?**

- Proposed a spatiotemporal prediction and multi-modal fusion framework that actively freezes template updates (*η_k_* ≈ 0) to reject specular reflections.

**What is the implications of the main finding?**

- Achieved a 35% tracking error reduction and a 72.83 ms rapid re-identification latency, offering a highly robust solution for robotic visual sensing in complex environments.

**Abstract:**

Rear-view human tracking and re-identification remain critical challenges for robotic visual sensing in unmanned vehicles, particularly under adverse weather conditions and severe occlusion. Conventional deep learning models often suffer from feature contamination and trajectory drift under dynamic illumination. To overcome these bottlenecks, we propose a lightweight tracking framework driven by spatiotemporal prediction and multimodal feature fusion. Specifically, an ego-motion-aware Kalman prediction mechanism maintains temporal continuity during complete occlusions. Upon target reappearance, a multi-factor descriptor—fusing color histograms with geometric constraints—is employed within a dynamic Mahalanobis search region. This is coupled with a specular-reflection-penalized adaptive learning rate (*η_k_*) that actively freezes template updates during severe environmental degradation conditions. Evaluated on a custom Mecanum-wheeled robot, the proposed method achieves a peak precision of 94.2% and a tracking success rate of 93.4%. Extensive experiments in extreme rainy night scenarios demonstrate a 35% reduction in average tracking error, maintaining a Center Location Error (CLE) below 11 pixels. Furthermore, the system achieves a rapid target re-identification response of 72.83 ms during occlusion phases. Ultimately, this framework delivers a highly robust and real-time solution for autonomous navigation in complex dynamic environments.

## 1. Introduction

With the rapid proliferation of unmanned vehicles and autonomous robotic systems, robust visual perception has emerged as a cornerstone for safe navigation and intelligent human–robot interaction within complex environments. While the majority of current research efforts have predominantly focused on front-view perception, rear-view human tracking and re-identification in the context of robotic visual sensing remain critically underexplored. However, rear-view monitoring is indispensable for operational scenarios such as autonomous personal assistants, security patrol robots, and human–robot collaborative following [1,2,3,4]. In these applications, the ability to robustly track and re-identify a trailing human target—even when they temporarily fall behind, change pace, or navigate through crowded spaces—is fundamental to maintaining seamless interaction and preventing safety hazards. Consequently, developing highly reliable rear-view tracking frameworks tailored for human targets in unstructured environments is not only a pressing research frontier but also a fundamental requirement for the next generation of intelligent mobile robots.

Despite the impressive performance of modern tracking systems under controlled conditions, real-world open-world scenarios introduce a constellation of challenges that severely degrade system performance. First, adverse environmental conditions—particularly rainy nights—produce intense specular reflections, uneven illumination, and excessive sensor noise, which collectively corrupt appearance cues and significantly deteriorate feature discriminability. These complex visual artifacts undermine the stability of common appearance models. Second, in dynamic scenes populated with pedestrians and mobile obstacles, targets frequently undergo severe or even complete occlusions that disrupt continuity and confuse conventional trackers. Under such conditions, adversarial environments expose fundamental deficits in existing perception frameworks, leading to tracking failures that jeopardize real-time operations [5,6,7,8]. Taken together, these factors highlight a critical gap between idealized benchmark performance and the demands of real-world deployment.

Recent advances in deep learning–based object detection and tracking have achieved remarkable precision in standard benchmarks. However, several fundamental limitations prevent their seamless application to rear-view unmanned vehicles, particularly under adversarial conditions. First, while paradigm frameworks like DeepSORT excel in clear environments, they demonstrate significant instability when applied to densely populated or occlusion-heavy contexts [9,10]. Second, contemporary models are highly susceptible to feature contamination and trajectory drift. For instance, studies such as Sun et al. [11] on feature-enhanced Siamese networks have attempted to address low-light challenges by integrating dynamic templates for robust feature updates. However, these methods still implicitly rely on relatively stable illumination and frequently fail when visibility severely degrades (e.g., specular reflections on rainy nights). Third, addressing temporal discontinuity often comes at an unacceptable computational cost. Research by Wan et al. [12] proposed using 3D spatiotemporal graphs to reestablish fragmented target paths by integrating temporal motion priors. While effective for sustained tracking, such systems are prohibitively computationally intensive and poorly suited for onboard processing in edge-constrained scenarios, such as drones or small autonomous vehicles. Consequently, when a target briefly disappears and reappears, conventional lightweight mechanisms typically resort to blind global search strategies. This results in expensive feature matching across the entire image space (often exceeding 90 ms per frame [13]), compromising real-time responsiveness and making such approaches inadequate for mission-critical autonomous navigation.

Recent studies on robust target tracking have explored multimodal fusion and adaptive representation learning from different sensing perspectives. For example, transformer-based RGBT tracking frameworks [14] have demonstrated the effectiveness of combining cross-modal and spatiotemporal information for robust target association under challenging conditions. TIR-oriented studies [15] have further emphasized the importance of fine-grained feature modeling and template reconstruction for suppressing appearance degradation and improving temporal robustness. In addition, recent review literature on RGBT tracking [16] has highlighted the broader trend toward stronger fusion strategies, adaptive template mechanisms, and context-aware robustness in complex environments. These developments provide useful theoretical context for the present study.

While these state-of-the-art methodologies collectively establish a strong foundation for robust multimodal or template-aware tracking, the focus of our work differs from RGBT or pure TIR tracking. Rather than relying on visible–thermal cross-modal sensing, this paper addresses rear-view robotic target tracking in a visible-light sensing setting. In this constrained context, robustness must be achieved purely through algorithmic design to overcome severe illumination transitions, specular reflection, occlusion, and ego-motion constraints. The proposed method formulates a unified cue-level multimodal tracking framework by integrating complementary information from appearance similarity, geometric consistency, and spatiotemporal prediction, together with an adaptive reference-model update mechanism. In this sense, our framework can be viewed as a lightweight and deployment-oriented alternative to computationally intensive spatiotemporal fusion and template-management strategies, designed for real-time robotic perception under edge-computing constraints.

To mitigate the aforementioned limitations, this paper proposes a lightweight and robust rear-view target tracking and re-identification framework tailored for unmanned vehicles in complex dynamic environments. Specifically, we integrate a Kalman-driven spatiotemporal prediction mechanism that actively maintains the target’s kinematic continuity during severe occlusions. Rather than relying on computationally prohibitive global matching when the target reappears, our system triggers an active local search strategy. Within this dynamically constrained region, we construct a robust multi-factor descriptor by fusing quantized color histograms and geometric kinematics, ensuring high discriminability even under adverse weather conditions. Furthermore, an adaptive model update strategy with a dynamic learning rate (η) is introduced to selectively assimilate valid features and actively purge background noise, thereby preventing trajectory drift caused by specular reflections on rainy nights.

To avoid ambiguity in the terminology used in the title, we clarify the meanings of the two core concepts adopted in this work. First, “spatiotemporal prediction” in this paper does not merely refer to conventional Kalman-based state extrapolation or passive trajectory smoothing. Instead, it denotes a coupled mechanism that integrates motion continuity modeling, ego-motion-aware observation uncertainty adjustment, occlusion-triggered suspension of measurement updates, and covariance-constrained local target re-identification. Second, “multimodal fusion” in the present study does not specifically indicate multi-sensor fusion such as RGB-T tracking, but rather the joint use of heterogeneous yet complementary cues, including appearance similarity, geometric consistency, and motion-prediction information, within a unified rear-view human tracking framework. These terms are used to emphasize that the proposed method enhances robustness under illumination variation, specular reflection, occlusion, and robotic ego-motion by combining temporal prediction with multiple complementary tracking cues. The detailed formulations of these components are presented in Section 2.

The main contributions of this paper are summarized as follows:

1. A lightweight spatiotemporal prediction framework for severe occlusion: We propose an active tracking mechanism integrating a Kalman prior. Unlike traditional passive trackers, it strictly maintains temporal continuity and constrains the re-identification search space dynamically during complete target loss. This mathematically reduces the spatial search complexity from $O\left(W\times H\right)$ to $O\left(w^{\prime }\times h^{\prime }\right)$, fundamentally bypassing the severe latency of global blind searches.
2. A multimodal feature fusion and adaptive update strategy: We design a robust target descriptor combining appearance features (quantized HSV histograms) and spatiotemporal geometric constraints. Paired with a piecewise dynamic learning rate (*η_k_*) that actively freezes template updates during severe feature degradation, this strategy fundamentally prevents feature contamination and trajectory drift in exceptionally harsh environments (e.g., severe specular reflections on rainy nights).
3. Extensive real-world validation in adverse weather: We deploy and evaluate the proposed system on a custom Mecanum-wheeled unmanned robot. Experimental results demonstrate that the framework achieves a peak precision of 94.2% and a tracking success rate of 93.4%. Notably, in extreme rainy night scenarios, the system reduces the average tracking error by 35% (maintaining a Center Location Error below 11 pixels) and achieves a rapid re-identification latency of 72.83 ms during occlusion recovery, proving its high robustness and real-time engineering feasibility.

## 2. Materials and Methods

### 2.1. Overall System Architecture and Target Initialization

**Figure 1:**
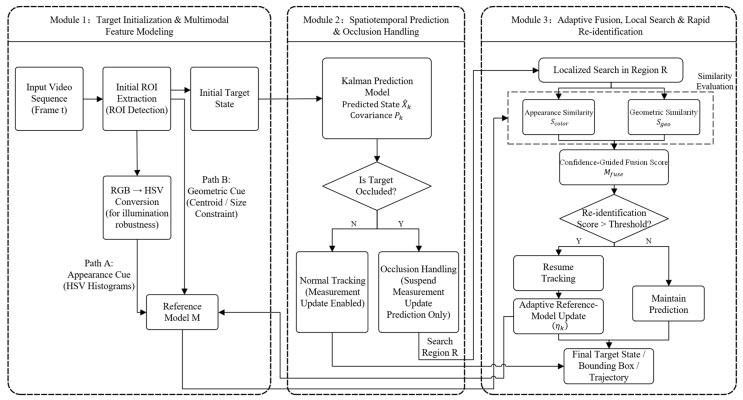
Detailed architecture of the proposed target tracking framework. The system consists of three coordinated modules: (1) target initialization and multimodal feature modeling; (2) spatiotemporal prediction and occlusion handling; and (3) adaptive fusion, local search, and rapid re-identification, integrating confidence-guided fusion $\left(M_{fuse}\right)$ and adaptive reference-model updating $\left(\eta_{k}\right)$.

To achieve high-robustness target tracking under complex dynamic environments (e.g., severe illumination variations and complete target occlusion) [17,18], this paper proposes an adaptive tracking framework based on multi-factor fusion and spatiotemporal prediction. The overall architecture of the proposed system is illustrated in Figure 1, which consists of three coordinated modules: (1) Target Initialization & Multimodal Feature Modeling; (2) Spatiotemporal Prediction & Occlusion Handling; and (3) Adaptive Fusion, Local Search & Rapid Re-identification.

During the system initialization phase, to ensure rapid response under the limited computational resources of mobile robots, a lightweight frame differencing method combined with adaptive threshold segmentation is employed to extract the initial region of interest (ROI) of the moving target. Upon acquiring the initial ROI, rather than utilizing the illumination-sensitive RGB color space, the system maps the visual data into the HSV (Hue, Saturation, Value) color space [19,20]. The HSV model decouples the brightness information (V component) from the chromaticity (H and S components). This transformation enables the system to effectively resist sudden external brightness fluctuations (as will be demonstrated in the rainy night scenarios in Section 3.8), thereby establishing a robust physical foundation for the subsequent multimodal feature modeling. Based on the initialized ROI, the system jointly constructs the initial target state and the reference model M by combining appearance and geometric cues, which serve as the basis for subsequent prediction and re-identification.

### 2.2. Robust Multimodal Feature Modeling

Relying on a single appearance feature is highly susceptible to failure in complex scenarios. Therefore, this paper constructs a multimodal feature model integrating color distribution and geometric constraints to enhance the discriminative power of the target against deformations and illumination fluctuations.

#### 2.2.1. Appearance Feature Based on HSV Histograms

The system extracts the color histograms of the target region in the H and S channels to serve as the appearance model. To quantify the appearance similarity between the current candidate target p and the reference target model p, the Bhattacharyya coefficient is introduced as a similarity metric [21,22]. The calculation is defined in Equation (1):

(1) $$\rho \left(p,q\right)=\sum_{u=1}^{m}\sqrt{p^{\left(u\right)}q^{\left(u\right)}}$$

where m represents the number of quantization levels in the color histogram (in this study, the H channel is quantized into 16 levels and the S channel into 8 levels). The appearance similarity score, denoted as $S_{color}$, is directly determined by the Bhattacharyya coefficient ρ. This metric effectively smooths local noise, ensuring that the system maintains a high feature matching rate even when the target undergoes non-rigid deformations.

#### 2.2.2. Geometric Topological Constraint Based on Centroids

In addition to color features, dynamic centroid constraints are introduced to filter out similar distractors in the background. Assuming that the bounding box region of the target in the k-th frame is $R_{k}$, its geometric centroid $\left(x_{c},y_{c}\right)$ calculation is simplified to the ratio of the zero-order to the first-order moments of the target contour. The geometric similarity score, $S_{geo}$, is dynamically computed by evaluating the Euclidean distance between the candidate bounding box and the predicted centroid. This dual-constraint mechanism of “color + geometry” effectively circumvents the tracking drift issues commonly encountered by single-feature trackers when facing background clutter with similar colors.

**Figure 2:**
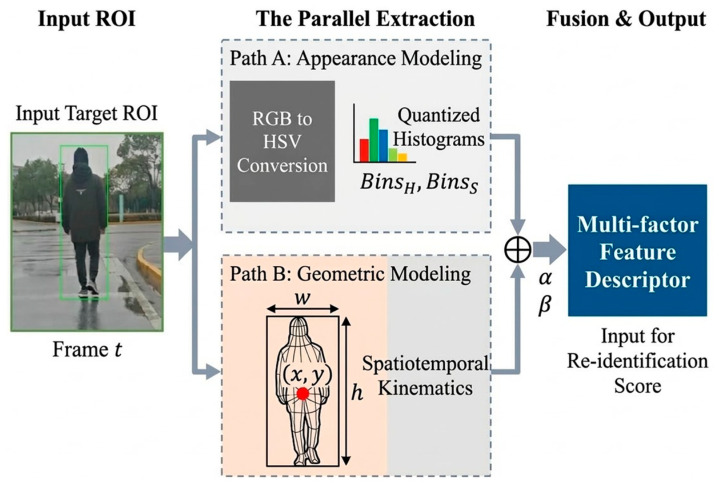
Conceptual schematic illustrating the parallel multimodal feature extraction and fusion process.

The proposed multimodal feature modeling process is conceptually illustrated in Figure 2. Raw input sequences undergo parallel processing: Path A detailing quantized color histograms ($Bins_{H},Bins_{S}$) for appearance descriptors, and Path B establishing spatiotemporal geometric constraints based on centroid coordinates $\left(x,y\right)$ and physical dimensions (w,h). These features are subsequently concatenated with adaptive weights (α,β) to form a comprehensive Multi-factor Feature Descriptor for high-fidelity re-identification.

Specifically, the adaptive weights (α,β) shown in Figure 2 are not static, but formulated as an environment-aware adaptive weighting mechanism to counteract severe feature degradation. The comprehensive multi-factor response score $M_{fuse}$ is mathematically defined in Equation (2):

(2) $$M_{fuse}=\alpha \left(t\right)\cdot S_{color}+\beta \left(t\right)\cdot S_{geo}$$

where $S_{color}$ and $S_{geo}$ represent the appearance and geometric similarity scores, respectively. To ensure robustness under complex lighting (e.g., specular reflections), the weights are modulated by the current target visibility confidence $C_{t}$, where α(t)=ω(t) and $\beta \left(t\right)=1-\omega \left(t\right)$, with $\omega \left(t\right)$ calculated using Equation (3):

(3) $$\omega \left(t\right)=\frac{1}{1+e^{-\gamma \left(C_{t}-\tau_{occ}\right)}}$$

This Sigmoid-based activation ensures that during severe feature degradation or occlusions (when $C_{t}$ drops below $\tau_{occ}$), the system automatically penalizes the unreliable appearance descriptor (α(t)→0) and relies heavily on the geometric kinematic constraints (β(t)→1).

### 2.3. Spatiotemporal Prediction via Kalman Constraints

To address tracking drift caused by rapid object motion or temporary occlusion, a linear discrete Kalman filter is employed to provide reliable spatiotemporal priors. The system models the target’s kinematics using a constant velocity assumption.

Let the system state vector at frame k be defined as $X_{k}={\left[x_{k},y_{k},v_{xk},v_{yk}\right]}^{T}$, where $\left(x_{k},y_{k}\right)$ denotes the centroid coordinates, and $\left(v_{xk},v_{yk}\right)$ represents the corresponding velocities along the x and y axes. The state transition model is formulated as Equation (4):

(4) $$X_{k}=AX_{k-1}+W_{k-1}$$

For the constant velocity kinematic model, assuming that the frame interval is Δt, the state transition matrix A and the measurement matrix H are explicitly defined in Equation (5):

(5) $$A=\left[\begin{array}{cccc} 1 & 0 & \Delta t & 0\\ 0 & 1 & 0 & \Delta t\\ 0 & 0 & 1 & 0\\ 0 & 0 & 0 & 1 \end{array}\right],H=\left[\begin{array}{cccc} 1 & 0 & 0 & 0\\ 0 & 1 & 0 & 0 \end{array}\right]$$

The process noise covariance Q and measurement noise covariance R are initialized empirically based on the camera frame rate and expected target dynamics.

Subsequently, the observation model is defined as Equation (6):

(6) $$Z_{k}=HX_{k}+V_{k}$$

where A and H are the state transition matrix and measurement matrix, respectively. $W_{k-1}$ and $V_{k}$ represent the process and measurement noises, assumed to be zero-mean Gaussian white noise.

Unlike static surveillance, rear-view tracking in unmanned vehicles introduces ego-motion-induced vibrations. To address this hardware constraint, we propose an ego-motion-aware dynamic observation noise model. The measurement noise covariance R is dynamically coupled with the vehicle’s real-time velocity vector $v_{ego,k}$, as expressed in Equation (7):

(7) $$R_{k}=R_{0}+\kappa \cdot ∥v_{ego,k}{∥}^{2}\cdot I$$

where $R_{0}$ is the base static noise and κ is the vibration coupling coefficient. This formulation ensures that the Kalman filter automatically suppresses unreliable visual measurements during high-speed or uneven maneuvers [23,24].

Crucially, this paper proposes an active occlusion-handling mechanism based on the Kalman constraints. During normal tracking conditions, the filter performs standard time updates (prediction) and measurement updates (correction) sequentially. However, when the target undergoes severe or complete occlusion (as verified in the spatial reasoning phase in Section 3.9), the target’s visual features become unreliable. In such scenarios, the measurement update is actively suspended. The system relies exclusively on the state transition model (A) to iteratively predict the target’s hidden trajectory, ensuring that a valid search region is continuously maintained even when the target is completely invisible [25,26].

**Figure 3:**
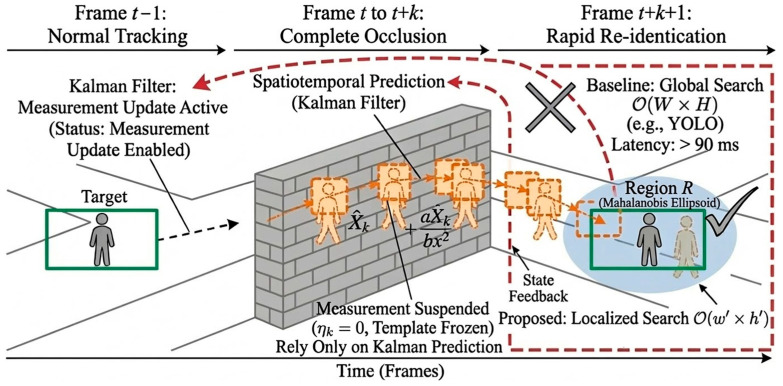
Schematic illustration of the active spatiotemporal prediction and rapid local search mechanism. The red dashed arrows indicate the spatiotemporal prediction path during occlusion, the orange dashed arrows and boxes denote predicted or candidate target positions, and the solid black arrows indicate the state-feedback and localized-search flow. The large “×” and “√” symbols indicate the non-adopted global-search strategy and the adopted localized-search strategy.

To intuitively demonstrate the active occlusion handling and rapid recovery mechanism, the state transition process is conceptually illustrated in Figure 3. During stable tracking, the observation model is continuously updated. However, when complete occlusion is detected, the measurement update is immediately suspended. The system then relies exclusively on the Kalman filter to infer the target’s hidden spatiotemporal trajectory (denoted by the orange dashed boxes). Upon the target’s reappearance, rather than executing a computationally expensive global search, the system triggers an active localized re-identification. Specifically, to overcome the $O\left(W\times H\right)$ computational bottleneck of conventional trackers, our mechanism leverages the Kalman prior covariance matrix $P_{k}$ to strictly constrain the multi-factor feature matching within a dynamic Mahalanobis ellipsoid R, defined as Equation (8):

(8) $$R=\left\{(x,y)|(Z-{\hat{Z}}_{k}{)}^{T}P_{k}^{-1}(Z-{\hat{Z}}_{k})\leq \chi_{p}^{2}\right\}$$

By gating the search region using the $\chi^{2}$ threshold, the spatial complexity drastically drops from $O\left(W\times H\right)$ to $O\left(w^{\prime }\times h^{\prime }\right)$, where $w^{\prime }\ll W$ and $h^{\prime }\ll H$. This rigorous spatial constraint avoids redundant background processing and contributes to efficient target recovery, which is evaluated in Section 3.

It should be emphasized that the “spatiotemporal prediction” mechanism proposed in this paper does not merely refer to conventional Kalman extrapolation, nor is it intended as a direct substitute for fully nonlinear motion-state modeling. Rather than explicitly fitting sharp-turn or rapid-acceleration dynamics through higher-order or nonlinear filtering, the present framework uses the Kalman prior as a lightweight temporal continuity constraint and augments it with ego-motion-aware observation-noise adaptation, occlusion-triggered suspension of unreliable measurement updates, and covariance-constrained local search during re-identification. Therefore, the distinction from standard Kalman-based motion modeling lies in the fact that prediction is not used as an isolated extrapolation module, but as part of an integrated robustness-oriented pipeline for maintaining target continuity and reducing recovery uncertainty under robot-induced motion perturbations.

### 2.4. Adaptive Fusion and Rapid Re-Identification Mechanism

Building upon the dynamically constrained local region R established in Section 2.3, the system executes a rapid re-identification process. As formulated in Equations (2) and (3) in Section 2.2, the weighting coefficients α and β dynamically balance the appearance and geometric features based on real-time visibility confidence. Furthermore, to maintain the purity of the reference model M (updated in Line 12 of Algorithm 1), a rigorous adaptive update strategy is required. A critical challenge in rainy night tracking is feature contamination from specular reflections, which characteristically manifest as extremely high Value (V) and low Saturation (S). We introduce a Specular Reflection Penalty ${(P}_{specular})$ for the target region $\Omega_{ROI}$, as calculated in Equation (9):

(9) $$P_{specular}=\frac{1}{\left|\Omega_{ROI}\right|}\sum_{(x,y)\in \Omega_{ROI}}\;I\left(V(x,y)>\tau_{v}\wedge S(x,y)<\tau_{s}\right)$$

The template update learning rate $\eta_{k}$ is then rigorously modeled as a piecewise exponential decay function, expressed as Equation (10):

(10) $$\eta_{k}=\left\{\begin{array}{cc} \eta_{base}\cdot exp(-\lambda \cdot P_{specular}), & if\;target\;is\;tracked\;securely\\ 0, & if\;occlusion\;persists \end{array}\right.$$

This ensures that the reference model M actively freezes $\left(\eta_{k}=0\right)$ during severe occlusions [27,28,29]. Furthermore, during extreme specular reflections, the exponential penalty effectively drives $\eta_{k}\approx 0$, thereby instantly rejecting contaminated features and fundamentally preventing trajectory drift.

Sensitivity to threshold selection can be understood directly from Equations (9) and (10). For a fixed target ROI, the specular-reflection penalty $P_{specular}$ is non-increasing with respect to the brightness threshold $\tau_{v}$, because a larger $\tau_{v}$ reduces the set of pixels satisfying $V(x,y)>\tau_{v}$. In contrast, $P_{specular}$ is non-decreasing with respect to the saturation threshold $\tau_{s}$, because a larger $\tau_{s}$ enlarges the set of pixels satisfying $S(x,y)<\tau_{s}$. Since the adaptive learning rate is modeled as $\eta_{k}=\eta_{base}exp(-\lambda P_{specular})$ in the normal tracked state, $\eta_{k}$ is correspondingly non-decreasing with $\tau_{v}$ and non-increasing with $\tau_{s}$. Therefore, smaller $\tau_{v}$ or larger $\tau_{s}$ makes the update mechanism more conservative and more likely to freeze template adaptation, whereas larger $\tau_{v}$ or smaller $\tau_{s}$ makes it more permissive and more likely to admit reflection-contaminated updates. The adopted threshold setting is chosen to balance these two effects, namely, suppressing contaminated updates while preserving sufficient adaptability under environmental disturbance. This interpretation also explains why excessively strict thresholds may lead to over-frequent freezing, whereas overly loose thresholds may weaken the rejection of reflection-induced feature contamination.

The complete logical flow of this adaptive re-identification and update phase is detailed in Algorithm 1.
**Algorithm 1.** Adaptive Multi-factor Target Re-identification.Input:$\mathrm{Current}\;\mathrm{frame}\;F_{k},\;$$\mathrm{Kalman}\;\mathrm{predicted}\;\mathrm{state}\;{\hat{X}}_{k},\;$Target reference model MOutput:$\mathrm{Updated}\;\mathrm{target}\;\mathrm{state}\;X_{k}$1:Define local search region R $\mathrm{centered}\;\mathrm{at}\;{\hat{X}}_{k}$2:$\mathrm{Extract}\;\mathrm{candidate}\;\mathrm{patches}\;\{C_{i}\}$within R via background subtraction3:$\mathrm{for}\;\mathrm{each}\;\mathrm{candidate}\;C_{i}\in \{C_{i}\}$ do4:$\mathrm{Compute}\;\mathrm{HSV}\;\mathrm{appearance}\;\mathrm{score}\;S_{color}\left(C_{i},M\right)$5:$\mathrm{Compute}\;\mathrm{geometric}\;\mathrm{shape}\;\mathrm{score}\;S_{geo}\left(C_{i},M\right)$6:$\mathrm{Calculate}\;\mathrm{combined}\;\mathrm{confidence}\;M_{fuse}=\alpha (t)\cdot S_{color}+\beta (t)\cdot S_{geo}$7:end for8:$\mathrm{Find}\;\mathrm{candidate}\;C^{\prime *}\;$$\mathrm{that}\;\mathrm{maximizes}\;M_{fuse}$9:$\mathrm{if}\;M_{fuse}\left(C^{*}\right)>Thresh_{reid}$ then10://Target successfully re-identified11:$\mathrm{Update}\;\mathrm{target}\;\mathrm{state}\;X_{k}\leftarrow C^{*}$12:$\mathrm{Update}\;\mathrm{reference}\;\mathrm{model}\;M\leftarrow (1-\eta_{k})M+\eta_{k}C^{*}$13:$\mathrm{Resume}\;\mathrm{Kalman}\;\mathrm{measurement}\;\mathrm{update}\;\mathrm{using}\;C^{*}$14:else15://Occlusion persists16:$\mathrm{Maintain}\;\mathrm{tracking}\;\mathrm{using}\;\mathrm{predicted}\;\mathrm{state}\;X_{k}\leftarrow {\hat{X}}_{k}$17:Suspend Kalman measurement update18:end if

To facilitate system replication and practical deployment, the core parameter configurations utilized across the aforementioned modules are summarized in Table 1.

## 3. Results

In this section, we analyze the experimental results obtained from the proposed target model update mechanism. The experiments were conducted under varying environmental conditions to evaluate the effectiveness, adaptability, and stability of the model during dynamic changes.

### 3.1. Dataset and Experimental Protocol

To rigorously evaluate the proposed tracking framework under complex real-world robotic sensing conditions, we constructed a custom real-world evaluation dataset using the proposed robotic platform. Because the proposed framework relies on explicit dynamic feature updating and Kalman-based motion modeling rather than end-to-end retraining on the collected sequences, this dataset is used strictly for evaluation rather than being divided into conventional training, validation, and testing subsets. All compared methods were evaluated on the same sequence set under consistent evaluation conditions to ensure a fair comparison.

As summarized in Table 2, the dataset comprises 51 challenging video sequences collected under three representative scenario categories, each designed to assess different aspects of tracking performance. Specifically, the dataset includes 26 nighttime post-rain reflection sequences, 9 outdoor dynamic-lighting sequences, and 16 indoor corridor sequences involving complete occlusion and reappearance events. Each sequence lasts approximately 20–30 s. The nighttime post-rain subset was recorded after rainfall had stopped, when wet road surfaces and strong streetlight reflections created severe specular interference. The outdoor dynamic-lighting subset captures abrupt brightness transitions as the target moves between direct sunlight and building-shadow regions. The indoor corridor subset contains complete occlusion–reappearance events caused by corner-induced target disappearance, which are used to evaluate target re-identification performance and recovery latency.

Furthermore, to ensure the reliability of the quantitative evaluation, the ground truth for all sequences was generated through manual frame-by-frame annotation using LabelImg (version 1.8.6), followed by consistency checking. Since the objective of this study is robotic target-following of a designated subject rather than generic multi-person tracking or recognition, the collected sequences mainly consist of repeated recordings of the same target subject under different environmental conditions. The detailed annotation protocol and the ground-truth generation process for different evaluation metrics are summarized in Table 3.

To further clarify the reliability of the evaluation, Table 3 summarizes the annotation protocol and the ground-truth generation process used for different metrics.

All compared methods were evaluated on the same annotated sequences under the same protocol to ensure fair comparison.

### 3.2. Hardware and Software Experimental Environment

This research employs a Raspberry Pi 4B single-board computer (Raspberry Pi Ltd., Cambridge, UK) as the core embedded computing platform. The hardware system comprises an omnidirectional Mecanum-wheeled chassis, an aluminum alloy frame, and a binocular stereo camera. The software environment is built upon Raspbian OS, with Python 3.9.18 (Python Software Foundation, Wilmington, DE, USA) utilized for the end-to-end development of the system.

At the perception layer, the YOLOv8n lightweight detection model is deployed for inference based on the PyTorch 1.12.0 framework. To address the constraints of embedded hardware, the ARM Neon instruction set is leveraged to optimize the floating-point computational performance of the Broadcom BCM2711 (Cortex-A72, 1.5 GHz) CPU, thereby ensuring the fulfillment of real-time processing requirements.

**Figure 4:**
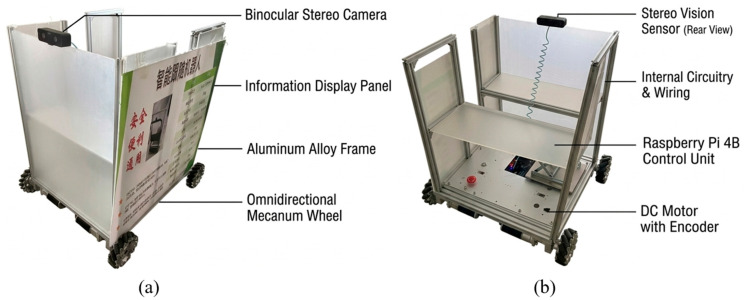
Physical prototype of the intelligent tracking robot: (**a**) Exterior design and overall structure; (**b**) Internal hardware integration layout. The Chinese text on the exterior panel is a generic safety warning.

Experimental Parameter Settings:

During the experiments, the binocular stereo camera is mounted at the top-front of the robot, with the acquisition resolution configured at 1280 × 720. The closed-loop control frequency of the system is stabilized at 30 Hz. The control layer implements a PID algorithm integrated with the kinematic model of the Mecanum wheels to achieve coordinated regulation of the robot’s linear velocity and yaw angle. All reported runtime-related results in this study, including the re-identification latency discussed later, were measured on the deployed Raspberry Pi 4B robotic platform rather than on a separate high-performance GPU workstation (see Figure 4).

### 3.3. Evaluation Metrics

Frame-level precision, recall, and F1-score are calculated based on the successful target localization results over a sequence of length T. Center-based localization error and temporal stability metrics are employed to reflect the rear-view robotic tracking performance under dynamic noise.

Let the center of the predicted bounding box at frame t be denoted by ${\hat{c}}_{t}=({\hat{x}}_{t},{\hat{y}}_{t})$, and the center of the ground-truth bounding box be $c_{t}=(x_{t},y_{t})$. The frame-wise center localization error $e_{t}$ is computed as the Euclidean distance:

(11) $$e_{t}=∥{\hat{c}}_{t}-c_{t}{∥}_{2}$$

The overall Center Location Error (CLE) over the sequence is calculated as:

(12) $$CLE=\frac{1}{T}\sum_{t=1}^{T}\;e_{t}$$

The Tracking Success Rate (TSR) is defined as the percentage of frames in which the target is successfully localized. Localization at frame t is considered successful if $e_{t}$ strictly falls below a distance threshold $\tau_{th}$. TSR is formulated as:

(13) $$TSR=\frac{1}{T}\sum_{t=1}^{T}\;I(e_{t}<\tau_{th})\times 100\%$$

I(⋅) is the indicator function. Given the 1280×720 acquisition resolution in our experiments, the distance threshold $\tau_{th}$ is uniformly set to 20 pixels across all compared methods.

The stability metric is defined as the standard deviation of the localization error over the successfully tracked frames. Let $S=\left\{t|e_{t}<\tau_{th}\right\}$ denote the set of successfully tracked frames, with $N_{succ}$ being the total number of frames in S. The stability is computed as:

(14) $$Stability=\sqrt{\frac{1}{N_{succ}}\sum_{t\in S}\;(e_{t}-\mu_{succ}{)}^{2}}$$

$\mu_{succ}$ is the mean CLE of the frames in S. A lower stability value indicates smaller temporal fluctuation of localization error and therefore smoother tracking performance.

For the indoor occlusion subset, the re-identification latency is strictly measured as the time elapsed from the exact frame the occluded target re-enters the field of view until the tracker successfully recovers the target ID. The reported re-identification latency was measured on the deployed Raspberry Pi 4B robotic platform and averaged across multiple occlusion-reappearance cycles.

### 3.4. Effectiveness of the Model Update Mechanism

To verify the performance of the proposed target model update mechanism, a comparative experiment was conducted involving the Kalman Filter, YOLOv8-based tracker, YOLOv11-based tracker, and the Proposed Method.

To ensure a fair comparison, all baseline models were evaluated under strictly identical hardware conditions and evaluation protocols. The YOLOv8- and YOLOv11-based baselines were both implemented in tracking-by-detection mode using the same standard BoT-SORT association framework. During the inference phase, the original 1280 × 720 video frames were resized to a detector input of 640 × 640 using standard letterbox preprocessing to preserve the aspect ratio. The resulting predicted bounding boxes were subsequently mapped back to the original image coordinates for quantitative evaluation. The traditional Kalman-filter baseline was implemented as a constant-velocity filtering method without appearance-based re-identification. No scenario-specific re-tuning was applied during the evaluation process, ensuring that all compared methods were tested on the same annotated sequences under identical conditions. The detailed implementation configurations and fairness protocols for all baseline methods are explicitly summarized in Table 4.

The evaluation metrics include Precision (%), Recall (%), F1-score (%), Stability, and Tracking Success Rate (%), and the corresponding results are summarized in Table 5.

**Figure 5:**
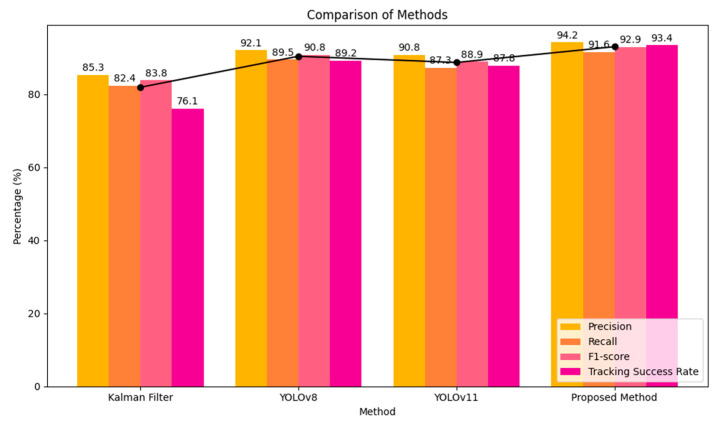
Graphical representation of performance metrics for various methods.

The experimental results, summarized in Table 5 and Figure 5, demonstrate the significant advantages of the proposed method: Detection Accuracy: The proposed method achieves a peak precision of 94.2%, outperforming the standard YOLOv8 (92.1%) and YOLOv11 (90.8%). This indicates superior feature extraction and target localization capabilities in complex backgrounds. Tracking Reliability: Our approach reaches a tracking success rate of 93.4%, which is 17.3 percentage points higher than the 76.1% achieved by the traditional Kalman Filter. This improvement highlights the effectiveness of the model update mechanism in preventing target loss. System Stability: In terms of tracking stability, the proposed method achieves the lowest stability value (4.7 px), whereas the Kalman Filter shows the largest temporal fluctuation (12.4 px). This ensures consistent performance during dynamic target changes and complex maneuvers of the Mecanum-wheeled chassis. This exceptional stability directly validates the effectiveness of the ego-motion-aware dynamic observation noise model ($R_{k}$) introduced in Section 2.3, which actively suppresses measurement jitter during omnidirectional movements.

It should be noted that the observed difference between YOLOv8 and YOLOv11 in this study is scenario- and configuration-dependent rather than a universal ranking between the two detector generations. Since both baselines were evaluated using the same BoT-SORT tracker, the same parameter configuration, the same detector input size, and the same evaluation protocol, the performance gap mainly reflects the stability of the detector outputs under the present rear-view robotic tracking setting. In our experiments, YOLOv11 exhibited more noticeable bounding-box jitter and larger frame-to-frame scale variation than YOLOv8, especially under illumination transitions and specular reflections. These fluctuations propagated into the downstream association stage and led to more frequent matching uncertainties, resulting in slightly inferior F1-score, stability, and tracking success rate compared with YOLOv8.

### 3.5. Ablation Analysis of Key Components

To further verify the contribution of the key modules in the proposed framework, an ablation analysis was conducted by selectively disabling one component at a time while keeping the remaining settings unchanged. The evaluated components include the geometric cue, the local-search constraint, the occlusion-triggered update-suspension mechanism, and the adaptive reference-model update strategy. All variants were evaluated on the same 51-sequence evaluation set under the same detector setting, initialization strategy, and evaluation protocol, without variant-specific re-tuning. As summarized in Table 6, the full model consistently achieves the best overall balance among all tested variants.

As summarized in Table 6, the full model achieves the best overall balance among all tested variants. Removing any key component results in measurable performance degradation, confirming the coordinated contribution of the proposed multi-module design.

Specifically, removing the geometric cue slightly reduces the re-identification latency (from 72.83 ms to 71.45 ms), likely due to the simplified matching process. However, this marginal speedup is accompanied by noticeable declines in Precision and TSR, indicating that geometric consistency plays an important role in discriminative target association under cluttered conditions.

When the local-search constraint is disabled, the re-identification latency increases markedly from 72.83 ms to 98.64 ms. This result highlights the importance of the covariance-constrained search region in reducing candidate ambiguity and maintaining efficient recovery.

Among all ablated variants, disabling the occlusion-triggered update-suspension mechanism leads to the most severe overall degradation, yielding the lowest Precision (88.6%), the lowest TSR (85.2%), and the worst Stability (8.4 px). This suggests that suppressing unreliable updates during target disappearance is important for preventing template contamination and cumulative trajectory drift.

Finally, removing the adaptive reference-model update strategy also degrades performance across all three tracking-quality metrics, showing that adaptive template evolution remains beneficial for maintaining robust target representation under environmental variation.

### 3.6. Dynamic Feature Evolution Analysis

The figure above demonstrates the application of the proposed target model update mechanism in response to dynamic environmental changes. The nine smaller images in the middle section show models generated from the template library under different environmental conditions. These images transition gradually, illustrating how the target model evolves under the influence of different data sources. The large image at the bottom represents the weighted average result of all templates after the model update, effectively showcasing the smooth transition between templates and the model’s ability to adapt to environmental changes.

In the experiment, the detection of drastic environmental changes and the rapid update mechanism of the target model played central roles. By dynamically adjusting the model over time, the proposed algorithm can quickly update the target model when a significant environmental change occurs, enabling it to respond to new environmental features. Over time, the model gradually incorporates new templates through weighted averaging, ultimately achieving effective adaptation to environmental shifts.

Moreover, during the transition of each model, the process is visually represented by arrows and color changes, indicating the gradual elimination of old models and the introduction of new ones. In this process, old models are shown in gray and represented by arrows flying out, signifying their failure in the current environment; the new models, in contrast, exhibit strong color transitions, indicating their rapid adaptation to the environmental change.

**Figure 6:**
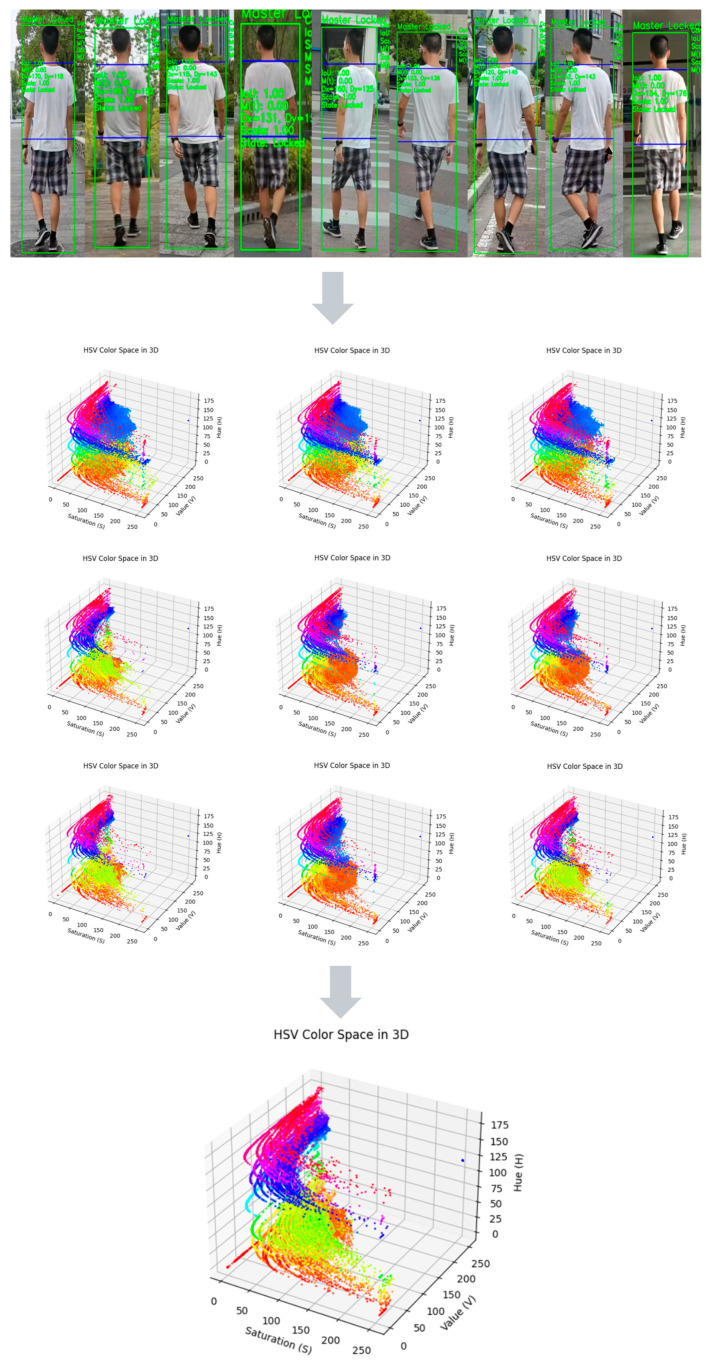
Application of the target model update mechanism from the model library under dynamic environmental changes. The upper image strip shows representative target observations, the nine middle 3D HSV plots visualize template-library feature models under different environmental conditions, and the bottom plot shows the updated fused model. The multicolored dots and curves in the 3D plots indicate HSV feature distributions under different environmental conditions, and the gray arrows indicate the update and aggregation flow toward the final fused model.

This experiment demonstrates that the proposed method not only effectively detects and responds to environmental changes but also maintains high stability and accuracy throughout the evolution of the target model. It highlights the adaptability and practicality of the model update mechanism in dynamic environments (see Figure 6).

### 3.7. Verification of the Model Update Mechanism

To validate the robustness and stability of the adaptive model update mechanism proposed in this paper under complex environmental changes, comparative experiments were conducted in scenarios involving dynamic lighting changes and complex background conditions.

The experiments selected the classical Kalman filter, the YOLOv8 tracking model, and the proposed Prediction + Adaptive Update Mechanism for performance comparison. The main focus of the evaluation was the tracking error and dynamic response characteristics of the learning rate under conditions where the target is not occluded but external environmental changes occur.

**Figure 7:**
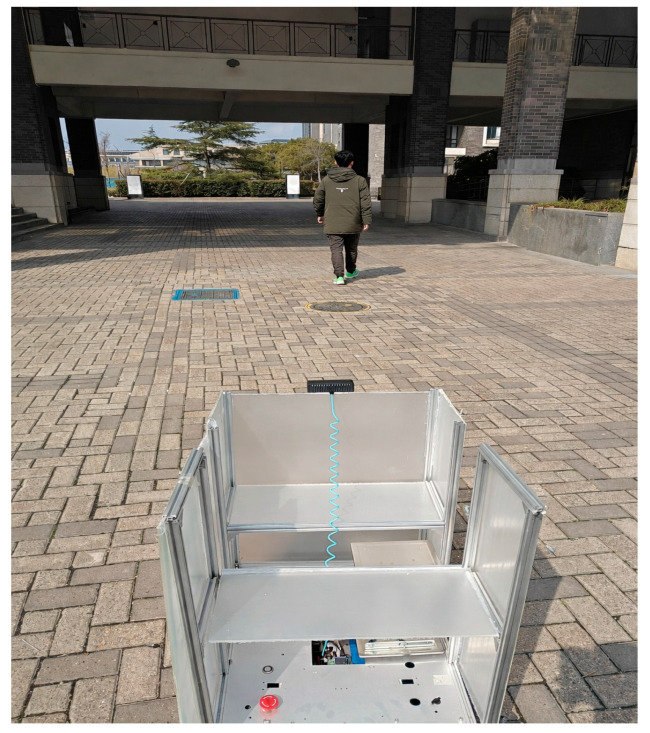
Real-time data acquisition scene using the mobile robot platform.

The camera viewpoint in Figure 7 illustrates the real-time data acquisition process in the experimental scene, including the target tracking performance under varying lighting conditions. The experimental scene simulated different environmental lighting changes (such as transitions from bright to dim, and strong light reflections) to test the system’s adaptability under dynamic lighting changes, and to evaluate the performance of different methods.

To ensure a rigorous and fair evaluation of continuous tracking performance, the state-of-the-art deep learning models (YOLOv8 and YOLOv11) evaluated in this study were strictly deployed in tracking-by-detection mode using the BoT-SORT association framework [30]. Consequently, they serve as representative end-to-end tracking baselines rather than mere frame-by-frame object detectors, providing a reliable benchmark for evaluating trajectory stability.

#### Analysis of Tracking Errors Under Lighting Variation

The evaluation is progressively conducted in two phases: initially verifying the baseline visual adaptability under regular shadow transitions, followed by a deep quantitative analysis of the tracking error and learning rate mechanisms under extreme disturbances.

**Figure 8:**
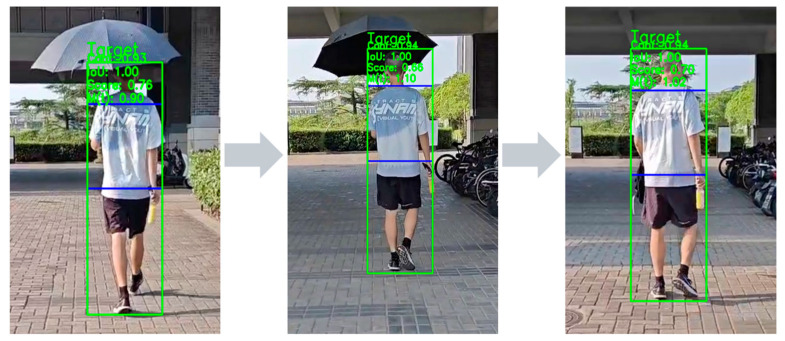
Demonstration of target motion image acquisition under varying lighting conditions in the experimental scene, serving as the foundation for the analysis of subsequent experimental results. The green bounding boxes and overlaid status labels indicate the target localization results generated by the tracking system. The blue horizontal lines are auxiliary tracking-visualization references, and the gray arrows indicate the temporal progression across frames.

As illustrated in Figure 8, we first evaluated the system in a common outdoor scenario where the target transitions from bright direct sunlight into a dense shadow. During this process, the overall illumination on the target drops abruptly, significantly altering the visual appearance of the clothing features. However, the proposed adaptive update mechanism smoothly integrates the newly darkened features into the tracking template library. As demonstrated by the green bounding boxes, the system maintains a tight and precise lock on the target throughout the entire illumination transition, exhibiting no noticeable scale distortion or trajectory drift. This baseline qualitative test confirms that the algorithm can effortlessly handle routine lighting variations in daily operations, establishing a solid foundation for the subsequent quantitative mechanism analysis and extreme stress tests.

### 3.8. Robustness and Adaptive Mechanism Analysis in Extreme Rainy Scenarios

Building upon the aforementioned mechanism analysis, this section evaluates the performance boundaries and ultimate robustness of the system by introducing a highly challenging rainy night scenario under streetlights. This environment presents severe local overexposure and specular ground reflections. Through a micro-level qualitative comparison across multiple frames, we comprehensively showcase the superior anti-interference capability of the proposed method when confronted with high-frequency false feature inductions.

**Figure 9:**
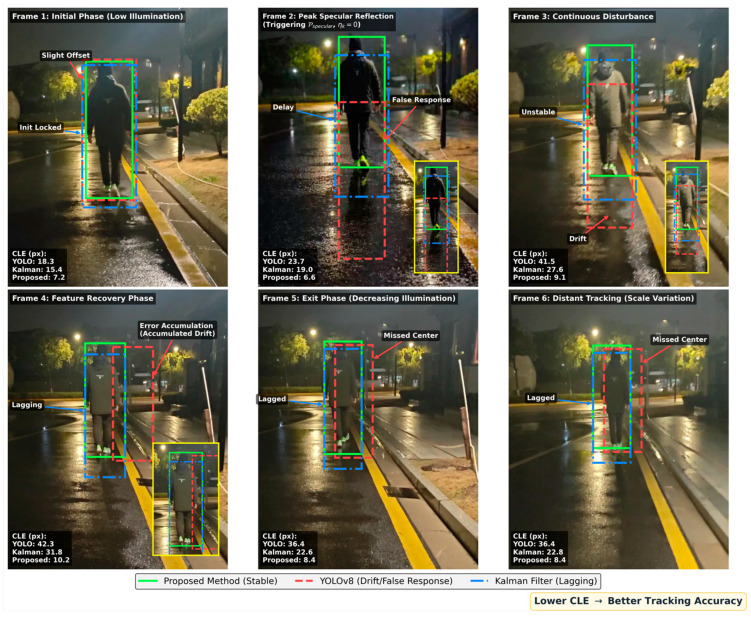
Qualitative tracking performance comparison in an extreme rainy night scenario with dynamic streetlights and specular reflections.

To further investigate the system’s dynamic response, Figure 9 presents a qualitative comparison of tracking sequences in a highly challenging rainy night scenario. This environment features low overall contrast coupled with severe specular reflections from wet surfaces under dynamic streetlights.

As shown in Frame 2 (Peak Specular Reflection), the standard YOLOv8-based tracker suffers from severe detection drift and template contamination. The specular reflections on the wet ground create false target features, causing the bounding box to improperly expand and shift towards the light source. Consequently, even as visibility relatively improves in Frame 4 (Feature Recovery Phase), the accumulated errors cause continued trajectory deviation, with the Center Location Error (CLE) peaking at 42.3 pixels. The Kalman Filter, while smoothing the trajectory, exhibits significant temporal lag (delay) across the sequence (e.g., Frames 4–6), as it relies primarily on linear motion momentum rather than real-time feature adaptation.

In stark contrast, the Proposed Method maintains a precise and stable lock throughout the entire sequence. By leveraging the adaptive model update mechanism, the system actively regulates the template learning rate. Specifically, as analyzed in Section 2.4, the Specular Reflection Penalty $\left(P_{specular}\right)$ rigorously identifies the high-Value and low-Saturation properties of the wet ground reflections. This triggers the piecewise function to instantly freeze the template $\left(\eta_{k}=0\right)$ during severe degradation in Frame 2. This mechanism effectively shields the tracking window from historical template contamination, ensuring robust recovery in subsequent frames (e.g., Frame 4) and keeping the CLE consistently below 11 pixels. This visual evidence strongly corroborates the quantitative stability metrics presented earlier, demonstrating the framework’s superior capability to manage complex environmental interference.

**Figure 10:**
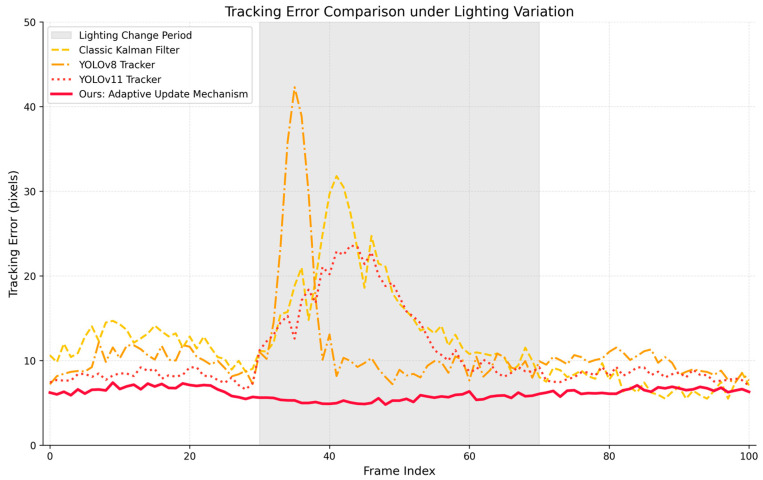
Continuous tracking error comparison over a 100-frame sequence in the extreme rainy night scenario.

Quantitative Error Analysis. To further quantify the visual performance observed in Figure 9 and Figure 10, this section plots the continuous tracking error over a 100-frame sequence. It is crucial to note that the “Lighting Change Period” (gray shaded area) in the graph precisely corresponds to the severe disturbance and feature recovery phases (Frames 2–4) shown in the qualitative sequence.

From the results, it can be observed that the classic Kalman filter experiences a significant increase in error during this disturbance phase, accompanied by noticeable drift. The YOLO trackers (YOLOv8 and YOLOv11) exhibit amplified error fluctuations, reflecting instability when faced with sudden environmental changes. In contrast, the proposed method maintains a highly stable error curve throughout the entire process, achieving the lowest error even during the severe disturbance phase, with an approximate reduction in average error of 35%.

These results demonstrate that the proposed adaptive update mechanism effectively mitigates the impact of external disturbances on recognition outcomes. It enables the system to maintain high-precision target tracking without the need for re-detection. Ultimately, the system achieves a “smooth update–no drift” characteristic during feature updates, significantly enhancing the overall robustness of the tracking framework.

**Figure 11:**
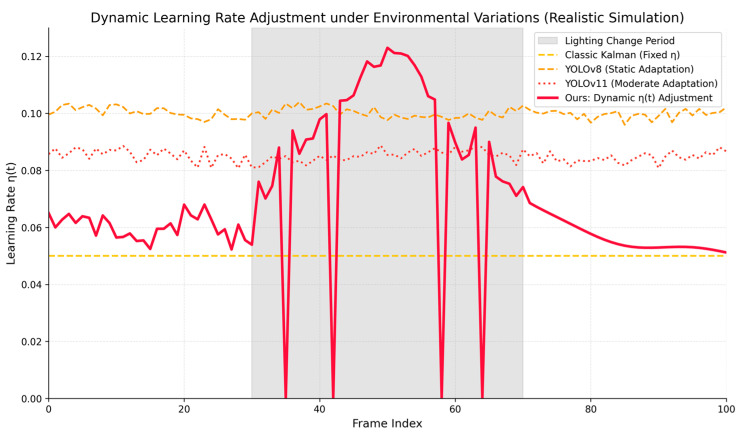
Dynamic adjustment of the learning rate η(t), responding to environmental disturbances during the rainy night sequence.

Underlying Adaptive Mechanism. The fundamental mechanism enabling this exceptional stability is revealed in Figure 11. The graph tracks the dynamic learning rate, η(t), corresponding to the same sequence. From the figure, it can be observed that during the disturbance phase (gray shaded area), the classic Kalman filter keeps its learning rate constant, leading to insufficient updating capability. YOLOv8 and YOLOv11, while showing some tracking state fluctuations, exhibit a slow adjustment process unable to quickly adapt to abrupt visual changes.

In contrast, the proposed method (red solid line) exhibits a highly sophisticated dual-layered adaptive mechanism. At a macro level, upon entering the “Lighting Change Period” (the globally illuminated zone under the streetlight), the baseline learning rate appropriately increases to rapidly adapt to the target’s newly emerging bright appearance features.

However, at a micro level, the red curve is not smooth; it is characterized by sharp, instantaneous downward dips during this disturbance phase. These sudden drops are the direct manifestation of the Specular Reflection Penalty $\left(P_{specular}\right)$ actively intervening. Whenever severe local degradation occurs (e.g., the intense wet ground reflections in Frame 2 of Figure 9), the mechanism aggressively suppresses the learning rate $\left(\eta_{k}=0\right)$ to prevent the template from incorporating contaminated puddle features. Once the environment stabilizes (after Frame 70), it quickly executes a smooth decay, effectively preventing overfitting to temporary noise. This decoupled adaptation proves that the algorithm can simultaneously embrace valid macroscopic illumination changes while rigorously rejecting microscopic environmental noise.

### 3.9. Trajectory Prediction and Target Re-Identification Analysis Under Occlusion

To validate the performance of the proposed prediction constraints and multi-factor fusion mechanism under complex scenarios involving target loss, this section conducts a comprehensive experimental analysis focusing on qualitative occlusion handling, trajectory prediction accuracy, and re-identification efficiency.

**Figure 12:**
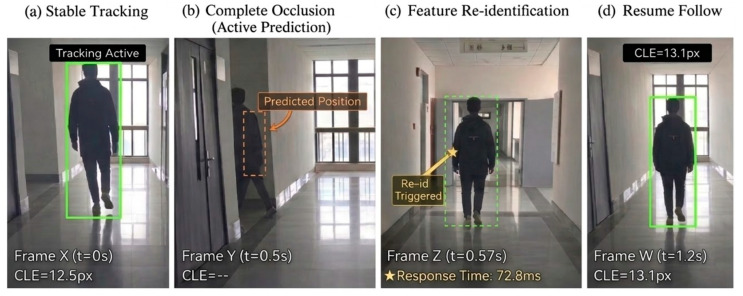
Qualitative tracking sequence demonstrating complete target occlusion handling in a real-world indoor corridor environment: (**a**) Stable tracking; (**b**) Complete occlusion (active prediction); (**c**) Feature re-identification; (**d**) Resume follow.

Qualitative Occlusion Handling Analysis. To visually demonstrate the system’s behavior under visibility loss, Figure 12 captures a representative sequence of the mobile robot handling complete occlusion in an indoor corridor. As shown in Figure 12b, when the target is fully obscured by the corridor wall, standard detection modules would typically fail. However, the proposed framework switches to an active prediction mode, maintaining an estimated trajectory (represented by the orange dashed box) [31]. This “blind-tracking” ensures that the system remains centered on the target’s hypothesized position. Upon target reappearance in Figure 12c, the system achieves rapid re-identification, with the trajectory ultimately resuming stable tracking in Figure 12d with a low CLE of 13.1 px. This qualitative evidence provides a strong foundation for the subsequent quantitative analysis.

**Figure 13:**
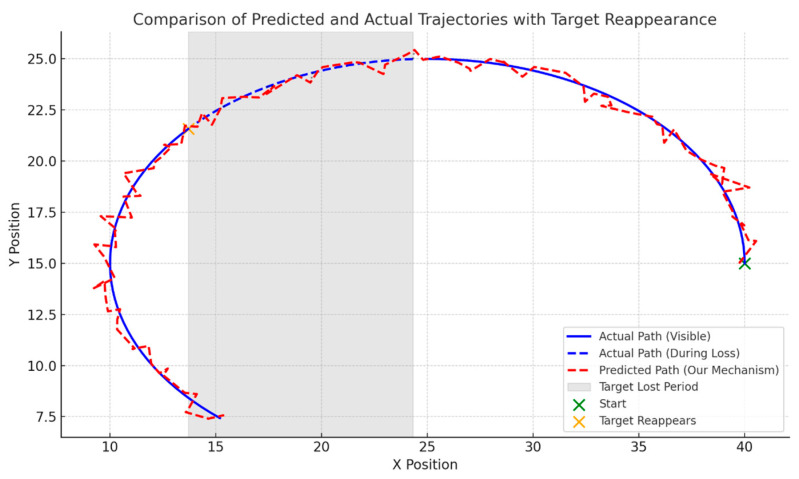
Comparison of predicted and actual trajectories during the target loss and reappearance process.

Trajectory Prediction Accuracy during Target Loss. Building upon the qualitative evidence in Figure 12 and Figure 13, this section illustrates the quantitative trajectory comparison between the proposed prediction mechanism and the ground truth during a target loss and reappearance event. The actual motion trajectory is denoted by the blue curve, where dashed segments represent the ground truth path during periods of occlusion. The estimated motion trajectory, derived from the prediction constraints, is depicted by the red dashed line. During the target’s invisibility phase (gray shaded area), the predicted trajectory preserves the motion trend via the Kalman state transition. Although a natural kinematic deviation accumulates during the prolonged invisibility phase, the prediction strictly bounds the target’s reappearance space, setting the stage for the instant position correction seen upon reappearance. This confirms that the model preserves trajectory continuity and effectively follows motion trends even in the absence of real-time observation data. Upon target reappearance (yellow marked point), the predicted path aligns precisely with the actual position, validating the high precision of the prediction model in spatial position estimation. Furthermore, by proactively predicting the target’s temporal position during the loss phase, the system significantly reduces re-identification latency overhead upon reappearance.

**Figure 14:**
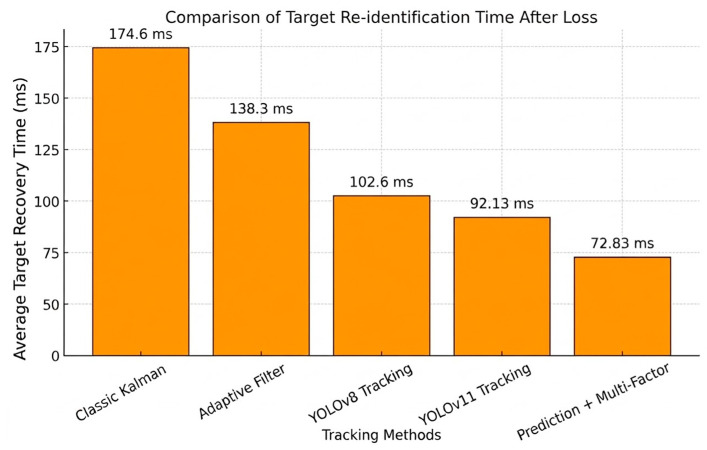
Comparison of average re-identification latency during the target re-identification phase after target loss for different tracking algorithms.

Target Re-identification Efficiency. High-precision spatial prediction directly translates to significantly reduced recovery latency. Figure 14 quantitatively compares the average re-identification latency during the target re-identification phase across various tracking frameworks, including the classic Kalman filter, improved adaptive filtering, deep learning detectors (YOLOv8, YOLOv11), and our proposed mechanism. The results indicate that traditional Kalman filters exhibit the highest delay (approx. 175 ms) due to their inherent inability to handle nonlinear motion and appearance variations during occlusion. While adaptive filtering reduces lag to 138 ms by dynamically adjusting noise parameters, it remains insufficient for high-speed robotic tasks. Deep learning models (YOLOv8, YOLOv11) leverage end-to-end feature extraction for accuracy, but are constrained by complex network inference times, resulting in response latencies between 90 and 105 ms.

In stark contrast, the proposed Prediction + Multi-Factor Mechanism integrates spatiotemporal constraints with multimodal appearance features to achieve high-precision position alignment immediately upon target reappearance by dynamically weighting and filtering candidate boxes within the predicted region (as demonstrated in the spatial reasoning of Figure 12 and Figure 13). By strictly confining the multi-factor feature matching to the dynamic Mahalanobis ellipsoid R, the spatial complexity is mathematically reduced to $O(w^{\prime }\times h^{\prime })$, enabling the system to complete re-identification in merely 72.83 ms. This represents an efficiency improvement of approximately 20.95% compared to the state-of-the-art YOLOv11-based tracker [32]. Such a significant reduction in latency highlights the system’s superior real-time performance and robustness in maintaining target continuity under challenging conditions.

### 3.10. Overall Performance Comparison

**Figure 15:**
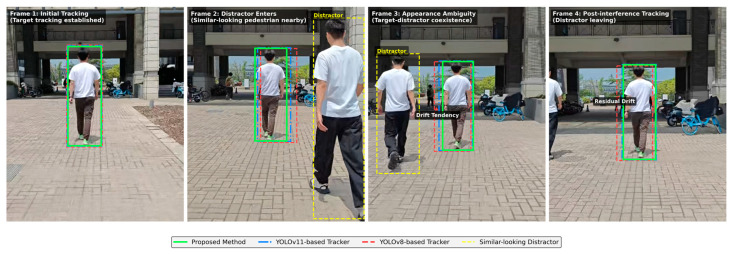
Qualitative comparison in a cluttered outdoor sequence with a similar-looking pedestrian distractor. The sequence was captured by the stereo camera mounted on the deployed robotic platform. Frame 1 shows the initial stable tracking state. In Frames 2 and 3, a visually similar pedestrian enters the field of view and coexists with the designated target, during which the YOLOv8- and YOLOv11-based trackers exhibit looser localization and drift tendency. In Frame 4, residual localization offset can still be observed in the baseline trackers, whereas the proposed method maintains a more stable target identity throughout the sequence.

Before summarizing the overall performance, we further include a representative qualitative case under cluttered outdoor conditions involving a similar-looking pedestrian distractor. Since the proposed framework performs cue-level multimodal fusion on visible-light robotic input, this qualitative comparison follows the same onboard visible-light input setting and baseline trackers as the quantitative evaluation. Figure 15 presents a rear-view tracking sequence in which a visually similar pedestrian enters the field of view and temporarily coexists with the designated target, thereby introducing potential identity ambiguity in a cluttered outdoor scene.

As shown in Figure 15, all compared trackers can initialize the target in the initial tracking frame. When the similar-looking pedestrian enters and coexists with the target in Frames 2 and 3, the YOLOv8- and YOLOv11-based trackers show different degrees of localization drift and residual offset, indicating increased association ambiguity under distractor interference. In contrast, the proposed method maintains a more consistent bounding box around the original target throughout the sequence.

To provide an overall quantitative comparison, this subsection summarizes the performance of all evaluated tracking methods based on Table 5. The Kalman-filter baseline exhibits the lowest tracking success rate (76.1%) and the largest Stability value (12.4 px), indicating its vulnerability to dynamic occlusions. While the YOLOv8- and YOLOv11-based trackers achieve relatively high precision (92.1% and 90.8%), they are susceptible to prolonged environmental disturbances (e.g., specular reflections), leading to larger temporal fluctuations in localization compared to the proposed method.

In contrast, the proposed Prediction + Multi-Factor mechanism achieves the best overall tracking performance. By utilizing the adaptive learning-rate mechanism ($\eta_{k}$) and constraining local searches within the Mahalanobis ellipsoid, the proposed method maintains more stable tracking under the tested disturbances. Consequently, as reported in Table 5, the proposed method attains the highest Precision (94.2%) and Tracking Success Rate (93.4%), along with the lowest Stability value (4.7 px). These quantitative results show that the proposed framework provides the most stable and reliable tracking performance among the compared methods under complex rear-view conditions.

## 4. Conclusions

In this paper, we proposed a lightweight and highly robust rear-view human tracking and re-identification framework tailored for robotic visual sensing in unmanned vehicles operating in complex, open-world environments. To overcome the critical vulnerabilities of conventional deep learning models when faced with adverse weather (e.g., rainy nights) and severe occlusions, our approach seamlessly integrated a Kalman-driven spatiotemporal prediction mechanism with a multimodal feature fusion strategy. By strictly confining the re-identification search space within a dynamic Mahalanobis ellipsoid and utilizing a robust descriptor (combining quantized HSV histograms and geometric kinematics), the system successfully decoupled target appearance from environmental noise. Furthermore, the introduction of a rigorous adaptive update strategy—driven by a Specular Reflection Penalty and a piecewise learning rate—effectively prevented trajectory drift by proactively freezing invalid feature assimilations.

Extensive real-world experiments conducted on a custom Mecanum-wheeled robot comprehensively validated the superiority of the proposed framework. The system achieved a peak precision of 94.2% and a tracking success rate of 93.4% without requiring large-scale end-to-end retraining on massive annotated datasets. Notably, under extreme rainy night conditions, our method reduced the average tracking error by 35%—maintaining a Center Location Error (CLE) below 11 pixels. During complete occlusion phases, the active local search mechanism enabled a rapid re-identification latency of 72.83 ms, bridging the critical gap between robustness and real-time edge computing constraints.

Ultimately, this research delivers a highly reliable, computationally efficient solution for intelligent human–robot interaction and autonomous tracking. Future work will focus on extending this framework to multi-target rear-view tracking scenarios and exploring its deployment on aerial platforms (UAVs) for cross-view collaborative perception.

## Figures and Tables

**Table 1:** Core Parameter Configurations of the Proposed Tracking System.

Module	Parameter Description	Symbol	Value/Setting
Initialization	Frame differencing threshold	$Thresh_{diff}$	25
	ROI morphological kernel size	$K_{morph}$	5×5
Feature Modeling	HSV Hue channel quantization	$Bins_{H}$	16
	HSV Saturation channel quantization	$Bins_{S}$	8
Kalman Filter	Time step interval (ms)	Δt	33 (approx. 30 fps)
	Mahalanobis gating threshold	$\chi_{p}^{2}$	9.488
Re-identification & Update	Re-identification triggering threshold	$Thresh_{reid}$	0.75
	Occlusion confidence threshold	$\tau_{occ}$	0.60
	Specular Value threshold	$\tau_{v}$	220
	Specular Saturation threshold	$\tau_{s}$	45
	Base template learning rate	$\eta_{base}$	0.02

**Table 2:** Summary of the custom testing dataset and evaluation purposes.

Scenario Category	Total Sequences	Approx. Duration per Sequence	Primary Tracking Challenges	Evaluation Purpose & Metrics
Post-rain nighttime reflection scenes	26	20–30 s	Specular reflections, low illumination, spurious appearance cues	Trajectory robustness, Center Location Error (CLE), stability
Dynamic Lighting	9	20–30 s	Abrupt illumination transitions, shadow boundary interference	Feature adaptation efficacy, learning rate dynamic response
Indoor Corridor	16	20–30 s	Complete target occlusion by structural obstacles	Re-identification latency, recovery success rate
Total	51	Approx. 17–25.5 min in total	Mixed real-world robotic sensing disturbances	Overall performance comparison

**Table 3:** Annotation protocol and ground-truth generation process.

Item	Description
Tracking Target	A designated rear-view human target subject.
Annotation Unit	Frame-by-frame target bounding box.
Annotation Tool	Open-source LabelImg software
Annotation Method	Manual frame-by-frame annotation using LabelImg, followed by consistency checking.
Ground-Truth for CLE Evaluation	Center coordinates extracted from the manually annotated bounding boxes.
Ground-Truth for Re-ID Evaluation	Frame indices corresponding to target disappearance, reappearance, and successful target re-locking events, manually identified from the annotated sequences.
Dataset Usage	Used exclusively for evaluation; not used for end-to-end model training.

**Table 4:** Baseline implementation details and fair-comparison protocol.

Method	Detector	Tracking/Association	Key Setting Principle	Fairness Protocol
Kalman Filter	—	Constant-velocity Kalman filtering	Fixed process/measurement setting	Same sequences, same evaluation criterion
YOLOv8-based tracker	YOLOv8	BoT-SORT	Fixed tracking-by-detection setting	Same sequences, same detector input size, no scenario-specific re-tuning
YOLOv11-based tracker	YOLOv11	BoT-SORT	Fixed tracking-by-detection setting	Same sequences, same detector input size, no scenario-specific re-tuning
Proposed Method	YOLOv8 + custom modules	Kalman + local search + adaptive update	Parameters listed in Table 1	Same sequences and same evaluation protocol

**Table 5:** Quantitative comparison of different algorithms.

Method	Precision (%)	Recall (%)	F1-Score (%)	Stability (Std. CLE, px) ↓	Tracking Success Rate
Kalman Filter	85.3	82.4	83.8	12.4	76.1
YOLOv8 + BoT-SORT	92.1	89.5	90.8	6.3	89.2
YOLOv11 + BoT-SORT	90.8	87.3	88.9	7.9	87.8
Proposed Method	94.2	91.6	92.9	4.7	93.4

Note: The down arrow (↓) indicates that lower values represent better performance.

**Table 6:** Ablation analysis of key components of the proposed framework.

Variant	Geometric Cue	Local Search Constraint	Occlusion-Triggered Update Suspension	Adaptive Reference-Model Update (*η_k_*)	Precision (%)	TSR (%)	Stability (px) ↓	Re-ID Latency (ms) ↓
Full model	✓	✓	✓	✓	94.2	93.4	4.7	72.83
*w*/*o* geometric cue	✗	✓	✓	✓	91.8	89.5	6.1	71.45
*w*/*o* local search constraint	✓	✗	✓	✓	92.4	90.2	5.8	98.64
*w*/*o* update suspension	✓	✓	✗	✓	88.6	85.2	8.4	75.34
*w*/*o* adaptive update	✓	✓	✓	✗	91.2	89.1	6.5	73.92

Note: Re-ID latency is measured on the indoor complete-occlusion subset only. Precision, TSR, and Stability are computed on the full 51-sequence evaluation set under the same evaluation protocol. √ and × indicate that the corresponding component is retained and removed, respectively, and ↓ indicates that lower values represent better performance.

## Data Availability

The data presented in this study are available on request from the corresponding author.
